# Chlorido{[2-(dicyclo­hexyl­phosphano­yl)eth­yl]bis­[2-(dicyclo­hexyl­phosphan­yl)eth­yl]phosphane}platinum(II) chloride dichloro­methane hemisolvate tetra­hydrate

**DOI:** 10.1107/S1600536808041615

**Published:** 2008-12-13

**Authors:** Timo Paul Rieckborn, Emine Karakoc, Jin He, Marc Heinrich Prosenc

**Affiliations:** a Institut für Anorganische und Angewandte Chemie der Universität Hamburg, Department Chemie, Martin-Luther-King-Platz 6, D-20146 Hamburg, Germany

## Abstract

The title compound, [PtCl(C_42_H_78_OP_4_)]Cl·0.5CH_2_Cl_2_·4H_2_O, crystallizes as a contact ion-pair with two close inter­molecular C—H⋯Cl^−^ contacts between CH acidic αH atoms of the phosphane ligand and the chloride anion. A chloride ligand together with three coordinating P ligand atoms create a slightly distorted square-planar coordination environment around the Pt^II^ center. An inter­molecular water O—H⋯Cl^−^ and water O—H⋯OP hydrogen-bond network completes the coordination around the anion. In addition, a disordered CH_2_Cl_2_ solvent mol­ecule cocrystallized within a hydro­phobic cavity spanned by the dicyclo­hexyl­phosphane ligands.

## Related literature

For related literature on Pt^II^ complexes, see: Brüggeller *et al.* (1992 ▶). For the structure of a related phosphane Pt^II^ complex with pendant P=O groups, see: Rieckborn *et al.* (2008 ▶). For selective activation of mol­ecular oxygen by platinum complexes, see: Aizawa *et al.* (2005 ▶); Poverenov *et al.* (2008 ▶). 
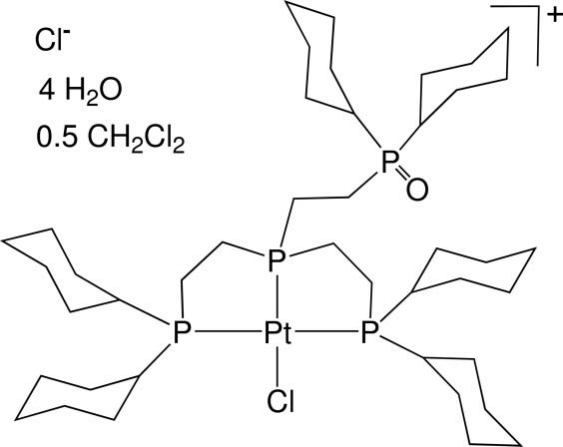


## Experimental

### 

#### Crystal data

[PtCl(C_42_H_78_OP_4_)]Cl·0.5CH_2_Cl_2_·4H_2_O
*M* *_r_* = 1103.39
Monoclinic, 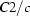

*a* = 20.3023 (18) Å
*b* = 28.077 (3) Å
*c* = 17.9581 (15) Å
β = 101.292 (2)°
*V* = 10038.6 (15) Å^3^
*Z* = 8
Mo *K*α radiation
μ = 3.12 mm^−1^
*T* = 100 (2) K
0.48 × 0.10 × 0.07 mm

#### Data collection

Bruker SMART APEX CCD area-detector diffractometer
Absorption correction: multi-scan (*SADABS*; Sheldrick, 1996 ▶) *T*_min_ = 0.694, *T*_max_ = 0.804
33754 measured reflections
11635 independent reflections
7471 reflections with *I* > 2σ(*I*)
*R*_int_ = 0.061

#### Refinement

*R*[*F* ^2^ > 2σ(*F* ^2^)] = 0.036
*wR*(*F* ^2^) = 0.063
*S* = 0.76
11635 reflections
544 parameters
18 restraints
H atoms treated by a mixture of independent and constrained refinement
Δρ_max_ = 1.86 e Å^−3^
Δρ_min_ = −1.61 e Å^−3^

### 

Data collection: *SMART* (Bruker, 2000 ▶); cell refinement: *SAINT* (Bruker, 2000 ▶); data reduction: *SAINT*; program(s) used to solve structure: *SHELXS97* (Sheldrick, 2008 ▶); program(s) used to refine structure: *SHELXL97* (Sheldrick, 2008 ▶); molecular graphics: *SHELXTL* (Sheldrick, 2008 ▶); software used to prepare material for publication: *SHELXTL*.

## Supplementary Material

Crystal structure: contains datablocks I, global. DOI: 10.1107/S1600536808041615/lh2731sup1.cif

Structure factors: contains datablocks I. DOI: 10.1107/S1600536808041615/lh2731Isup2.hkl

Additional supplementary materials:  crystallographic information; 3D view; checkCIF report

## Figures and Tables

**Table:** 

Pt1—P1	2.2078 (12)
Pt1—P2	2.3151 (11)
Pt1—P3	2.3215 (10)
Pt1—Cl1	2.3563 (11)

**Table:** 

P1—Pt1—P2	84.93 (4)
P1—Pt1—P3	86.57 (4)
P2—Pt1—P3	167.17 (4)
P1—Pt1—Cl1	176.99 (4)
P2—Pt1—Cl1	92.85 (4)
P3—Pt1—Cl1	95.26 (4)

**Table 2:** Hydrogen-bond geometry (Å, °)

*D*—H⋯*A*	*D*—H	H⋯*A*	*D*⋯*A*	*D*—H⋯*A*
O3—H3*C*⋯Cl2^i^	0.80	2.50	3.300 (3)	175
O3—H3*D*⋯Cl2	0.88	2.43	3.290 (4)	166
O4—H4*C*⋯O3	0.73	2.16	2.866 (4)	165
O4—H4*D*⋯O5^ii^	0.92	1.82	2.730 (4)	174
O5—H5*C*⋯O1	0.80	1.94	2.740 (4)	179
O5—H5*D*⋯O6	0.87	1.92	2.746 (5)	159
O6—H6*C*⋯Cl2	0.80 (4)	2.43 (4)	3.170 (4)	155 (8)
O6—H6*D*⋯Cl2^ii^	0.81 (7)	2.40 (7)	3.200 (4)	175 (9)
C3—H3*A*⋯Cl2	0.99	2.92	3.755 (4)	142
C5—H5*B*⋯Cl2	0.99	2.72	3.655 (4)	157

Symmetry codes: (i) 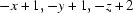; (ii) 
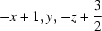
.

**Fig. 1.**
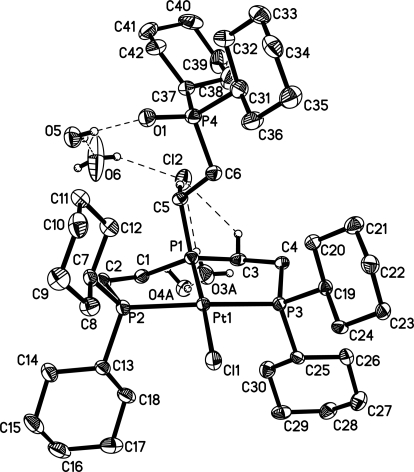
Representation of the Pt(II) complex with displacement ellipsoids at 50% probability. H atoms of the cyclohexyl and ethylene groups have been omitted for clarity. The hydrogen bonds between the anion and water molecules and the C—H groups respectively are depicted as dashed lines.

## supplementary crystallographic information

### Comment

Selective activation of molecular oxygen by platinum complexes is an active field of current research (Aizawa *et al.* 2005; Poverenov *et al.* 2008). Platinum^II^ complexes with pendant phosphane ligands are able to activate molecular oxygen revealing an oxidized phosphane group (Aizawa *et al.* 2005; Rieckborn *et al.* 2008). The title compund, [Pt(Cl)(C_42_H_76_OP_4_)]^+^ Cl^-^ * 4 H_2_O * 0.5 CH_2_Cl_2_ consists of a platinum(II) phosphane complex cation which crystallizes with a chloride anion as a contact ion pair including short CH···Cl contacts (2.72 Å and 2.92 Å). Further structural parameters are listed in Table 2. In addition four water molecules are connected *via* O–H···Cl2 (2.40 Å and 2.43 Å) bridges to the anion and *via* O–H···O=P (1.94 Å) bridges to the phosphane oxide group of the complex cation (Figure 1). The Pt^II^ centre is tetracoordinated revealing a slightly distorted square-planar coordination geometry (Brüggeller *et al.*, 1992). The phosphane oxide group of the ligand is not coordinated to the Pt^II^ centre in the solid state (Rieckborn *et al.*, 2008).

### Experimental

Unless otherwise stated all reactions were conducted under Schlenk techniques. Solvents were dried and stored under nitrogen.

Potassium tetrachloroplatinate(II) (215 mg, 0.52 mmol), was dissolved in 30 ml water and 30 ml e thanol was added. 367 mg (0.52 mmol) of Tris(2-(dicyclohexylphosphino)-ethyl)phosphane was dissolved in 15 ml dichloromethane and added to the reaction mixture. The colourless suspension was stirred at room temperature for 3 days. Afterwards the mixture was concentrated to small volume and a colourless solid precipitated. The precipitate was filtered and dried *in vacuo*. Single crystals were received by diffusion of n-hexane into a solution of the product in dichloromethane in air.

### Refinement

All non-hydrogen atoms were refined with anisotropic temperature parameters and H atoms were refined using a riding model with C—H distances set to 0.99 Å for aliphatic, and 0.80 Å for water O—H bonds. *U*_iso_(H) values were set to 1.2 *U*~eq~ for carbon bonded and to 1.5 *U*~eq~ for oxygen bonded H atoms of the parent atom. The dichloromethane molecule has been refined using half of a molecule of dichloromethane disordered over two sites with relative occupancies of s.o.f. 0.30 with the dichloromethane molecule lying on a twofold symmetry axis and the other dichloromethane molecule disordered close to the twofold rotation axis (s.o.f. 0.35). Thus, this site is occupied by one dichloromethane molecule. For the bonds C99—Cl4 and C99—Cl5 distances of 1.77 (1) were applied.

### Crystal data

**Table:**

[PtCl(C_42_H_78_OP_4_)]Cl·0.5CH_2_Cl_2_·4H_2_O	*F*(000) = 4560
*M**_r_* = 1103.39	*D*_x_ = 1.459 Mg m^−^^3^
Monoclinic, *C*2/*c*	Mo *K*α radiation, λ = 0.71073 Å
Hall symbol: -C 2yc	Cell parameters from 4947 reflections
*a* = 20.3023 (18) Å	θ = 2.3–23.8°
*b* = 28.077 (3) Å	µ = 3.12 mm^−^^1^
*c* = 17.9581 (15) Å	*T* = 100 K
β = 101.292 (2)°	Needle, colourless
*V* = 10038.6 (15) Å^3^	0.48 × 0.1 × 0.07 mm
*Z* = 8	

### Data collection

**Table:**

Bruker SMART APEX CCD area-detector diffractometer	11635 independent reflections
Radiation source: fine-focus sealed tube	7471 reflections with *I* > 2σ(*I*)
graphite	*R*_int_ = 0.061
φ and ω scans	θ_max_ = 28.1°, θ_min_ = 2.4°
Absorption correction: multi-scan (*SADABS*; Sheldrick, 1996)	*h* = −26→25
*T*_min_ = 0.694, *T*_max_ = 0.804	*k* = −20→37
33754 measured reflections	*l* = −23→20

### Refinement

**Table:**

Refinement on *F*^2^	Primary atom site location: structure-invariant direct methods
Least-squares matrix: full	Secondary atom site location: difference Fourier map
*R*[*F*^2^ > 2σ(*F*^2^)] = 0.036	Hydrogen site location: inferred from neighbouring sites
*wR*(*F*^2^) = 0.063	H atoms treated by a mixture of independent and constrained refinement
*S* = 0.76	*w* = 1/[σ^2^(*F*_o_^2^) + (0.0136*P*)^2^ + 2*P*] where *P* = (*F*_o_^2^ + 2*F*_c_^2^)/3
11635 reflections	(Δ/σ)_max_ = 0.003
544 parameters	Δρ_max_ = 1.86 e Å^−^^3^
18 restraints	Δρ_min_ = −1.61 e Å^−^^3^

### Special details

**Table:**

Geometry. All e.s.d.'s (except the e.s.d. in the dihedral angle between two l.s. planes) are estimated using the full covariance matrix. The cell e.s.d.'s are taken into account individually in the estimation of e.s.d.'s in distances, angles and torsion angles; correlations between e.s.d.'s in cell parameters are only used when they are defined by crystal symmetry. An approximate (isotropic) treatment of cell e.s.d.'s is used for estimating e.s.d.'s involving l.s. planes.
Refinement. Refinement of *F*^2^ against ALL reflections. The weighted *R*-factor *wR* and goodness of fit *S* are based on *F*^2^, conventional *R*-factors *R* are based on *F*, with *F* set to zero for negative *F*^2^. The threshold expression of *F*^2^ > σ(*F*^2^) is used only for calculating *R*-factors(gt) *etc*. and is not relevant to the choice of reflections for refinement. *R*-factors based on *F*^2^ are statistically about twice as large as those based on *F*, and *R*- factors based on ALL data will be even larger.

### Fractional atomic coordinates and isotropic or equivalent isotropic displacement parameters (Å^2^)

**Table:**

	*x*	*y*	*z*	*U*_iso_*/*U*_eq_	Occ. (<1)
Pt1	0.297423 (8)	0.640641 (7)	0.845227 (9)	0.01690 (5)	
P1	0.35938 (6)	0.57558 (4)	0.85196 (6)	0.0177 (3)	
P2	0.30418 (5)	0.64073 (5)	0.71803 (6)	0.0200 (3)	
P3	0.31646 (5)	0.63818 (5)	0.97692 (6)	0.0179 (2)	
P4	0.21292 (6)	0.45580 (5)	0.81651 (7)	0.0235 (3)	
Cl1	0.23492 (6)	0.71189 (4)	0.83402 (6)	0.0275 (3)	
Cl2	0.47682 (6)	0.45429 (5)	0.88578 (6)	0.0343 (3)	
O1	0.18711 (14)	0.46476 (11)	0.73396 (15)	0.0286 (8)	
C1	0.4140 (2)	0.58277 (16)	0.7839 (2)	0.0211 (10)	
H1B	0.4480	0.6077	0.8015	0.025*	
H1C	0.4376	0.5525	0.7784	0.025*	
C2	0.3701 (2)	0.59722 (16)	0.7074 (2)	0.0220 (11)	
H2A	0.3485	0.5684	0.6818	0.026*	
H2B	0.3990	0.6111	0.6747	0.026*	
C3	0.4059 (2)	0.56782 (16)	0.9477 (2)	0.0206 (11)	
H3A	0.4193	0.5340	0.9559	0.025*	
H3B	0.4472	0.5874	0.9553	0.025*	
C4	0.3630 (2)	0.58266 (15)	1.0055 (2)	0.0181 (10)	
H4A	0.3924	0.5872	1.0558	0.022*	
H4B	0.3307	0.5569	1.0101	0.022*	
C5	0.3191 (2)	0.51863 (15)	0.8243 (2)	0.0193 (10)	
H5A	0.3036	0.5185	0.7685	0.023*	
H5B	0.3531	0.4931	0.8371	0.023*	
C6	0.2592 (2)	0.50651 (16)	0.8612 (2)	0.0226 (11)	
H6A	0.2288	0.5344	0.8573	0.027*	
H6B	0.2756	0.4996	0.9157	0.027*	
C7	0.2295 (2)	0.62466 (16)	0.6469 (2)	0.0218 (11)	
H7A	0.2449	0.6176	0.5985	0.026*	
C8	0.1788 (2)	0.66504 (17)	0.6306 (2)	0.0280 (12)	
H8A	0.2004	0.6933	0.6126	0.034*	
H8B	0.1637	0.6739	0.6779	0.034*	
C9	0.1176 (2)	0.65002 (17)	0.5697 (2)	0.0323 (13)	
H9A	0.0840	0.6760	0.5625	0.039*	
H9B	0.1321	0.6449	0.5208	0.039*	
C10	0.0854 (2)	0.60483 (18)	0.5921 (3)	0.0345 (13)	
H10A	0.0488	0.5949	0.5499	0.041*	
H10B	0.0653	0.6113	0.6371	0.041*	
C11	0.1364 (2)	0.56447 (17)	0.6103 (2)	0.0309 (12)	
H11A	0.1147	0.5366	0.6292	0.037*	
H11B	0.1516	0.5548	0.5634	0.037*	
C12	0.1964 (2)	0.57966 (17)	0.6696 (2)	0.0246 (11)	
H12A	0.2299	0.5535	0.6776	0.030*	
H12B	0.1818	0.5853	0.7184	0.030*	
C13	0.3309 (2)	0.69867 (16)	0.6890 (2)	0.0211 (10)	
H13A	0.2932	0.7212	0.6914	0.025*	
C14	0.3418 (2)	0.70027 (17)	0.6068 (2)	0.0304 (12)	
H14A	0.3783	0.6780	0.6013	0.036*	
H14B	0.3003	0.6896	0.5721	0.036*	
C15	0.3600 (2)	0.75041 (18)	0.5843 (3)	0.0365 (13)	
H15A	0.3696	0.7496	0.5323	0.044*	
H15B	0.3213	0.7719	0.5839	0.044*	
C16	0.4207 (2)	0.76959 (18)	0.6388 (3)	0.0392 (14)	
H16A	0.4297	0.8027	0.6247	0.047*	
H16B	0.4606	0.7500	0.6356	0.047*	
C17	0.4080 (2)	0.76847 (17)	0.7204 (3)	0.0350 (13)	
H17A	0.4481	0.7806	0.7556	0.042*	
H17B	0.3697	0.7896	0.7240	0.042*	
C18	0.3925 (2)	0.71831 (16)	0.7436 (2)	0.0274 (12)	
H18A	0.3838	0.7187	0.7959	0.033*	
H18B	0.4317	0.6974	0.7429	0.033*	
C19	0.24466 (19)	0.63714 (16)	1.0238 (2)	0.0188 (9)	
H19A	0.2613	0.6268	1.0774	0.023*	
C20	0.1922 (2)	0.60065 (16)	0.9864 (2)	0.0240 (11)	
H20A	0.1757	0.6096	0.9327	0.029*	
H20B	0.2134	0.5689	0.9874	0.029*	
C21	0.1331 (2)	0.59805 (17)	1.0269 (3)	0.0285 (12)	
H21A	0.0992	0.5755	0.9997	0.034*	
H21B	0.1488	0.5857	1.0790	0.034*	
C22	0.1008 (2)	0.64619 (17)	1.0309 (2)	0.0282 (11)	
H22A	0.0644	0.6435	1.0602	0.034*	
H22B	0.0807	0.6571	0.9790	0.034*	
C23	0.1522 (2)	0.68244 (16)	1.0686 (2)	0.0222 (11)	
H23A	0.1697	0.6729	1.1219	0.027*	
H23B	0.1305	0.7140	1.0690	0.027*	
C24	0.2105 (2)	0.68604 (15)	1.0261 (2)	0.0187 (10)	
H24A	0.2438	0.7095	1.0518	0.022*	
H24B	0.1935	0.6973	0.9736	0.022*	
C25	0.3718 (2)	0.68629 (15)	1.0232 (2)	0.0179 (10)	
H25A	0.3494	0.7172	1.0060	0.021*	
C26	0.3821 (2)	0.68514 (16)	1.1102 (2)	0.0236 (11)	
H26A	0.3380	0.6873	1.1257	0.028*	
H26B	0.4033	0.6546	1.1293	0.028*	
C27	0.4265 (2)	0.72639 (18)	1.1448 (2)	0.0313 (13)	
H27A	0.4036	0.7569	1.1285	0.038*	
H27B	0.4334	0.7246	1.2008	0.038*	
C28	0.4940 (2)	0.72543 (18)	1.1210 (2)	0.0293 (12)	
H28A	0.5211	0.7531	1.1432	0.035*	
H28B	0.5185	0.6961	1.1406	0.035*	
C29	0.4853 (2)	0.72684 (16)	1.0354 (2)	0.0268 (12)	
H29A	0.5298	0.7243	1.0209	0.032*	
H29B	0.4652	0.7577	1.0163	0.032*	
C30	0.4402 (2)	0.68627 (17)	0.9986 (2)	0.0260 (11)	
H30A	0.4630	0.6555	1.0128	0.031*	
H30B	0.4330	0.6894	0.9427	0.031*	
C31	0.1451 (2)	0.44705 (17)	0.8681 (2)	0.0283 (12)	
H31A	0.1651	0.4376	0.9214	0.034*	
C32	0.0976 (2)	0.40718 (17)	0.8315 (3)	0.0326 (12)	
H32A	0.0847	0.4132	0.7762	0.039*	
H32B	0.1219	0.3764	0.8387	0.039*	
C33	0.0344 (2)	0.40347 (18)	0.8646 (3)	0.0312 (12)	
H33A	0.0038	0.3800	0.8347	0.037*	
H33B	0.0467	0.3914	0.9173	0.037*	
C34	−0.0026 (2)	0.45034 (18)	0.8652 (3)	0.0346 (13)	
H34A	−0.0411	0.4461	0.8909	0.042*	
H34B	−0.0202	0.4607	0.8124	0.042*	
C35	0.0443 (2)	0.48812 (18)	0.9063 (3)	0.0381 (14)	
H35A	0.0588	0.4790	0.9603	0.046*	
H35B	0.0202	0.5189	0.9043	0.046*	
C36	0.1059 (2)	0.49382 (18)	0.8701 (3)	0.0354 (13)	
H36A	0.1362	0.5179	0.8990	0.042*	
H36B	0.0914	0.5058	0.8176	0.042*	
C37	0.2716 (2)	0.40533 (16)	0.8290 (2)	0.0253 (11)	
H37A	0.3138	0.4179	0.8153	0.030*	
C38	0.2927 (3)	0.38674 (19)	0.9104 (3)	0.0433 (15)	
H38A	0.3095	0.4135	0.9448	0.052*	
H38B	0.2533	0.3727	0.9272	0.052*	
C39	0.3478 (2)	0.34906 (18)	0.9143 (3)	0.0403 (15)	
H39A	0.3881	0.3637	0.9005	0.048*	
H39B	0.3604	0.3369	0.9669	0.048*	
C40	0.3242 (2)	0.30842 (17)	0.8613 (3)	0.0388 (14)	
H40A	0.2854	0.2926	0.8768	0.047*	
H40B	0.3607	0.2847	0.8643	0.047*	
C41	0.3038 (3)	0.32640 (19)	0.7802 (3)	0.0386 (14)	
H41A	0.2868	0.2995	0.7463	0.046*	
H41B	0.3436	0.3398	0.7634	0.046*	
C42	0.2498 (2)	0.36445 (18)	0.7741 (3)	0.0355 (13)	
H42A	0.2082	0.3501	0.7852	0.043*	
H42B	0.2396	0.3769	0.7216	0.043*	
O3	0.57043 (15)	0.54710 (12)	0.94855 (17)	0.0400 (10)	
H3C	0.5603 (4)	0.54853 (12)	0.9894 (14)	0.060*	
H3D	0.5423 (10)	0.5255 (7)	0.9247 (8)	0.060*	
O4	0.71140 (15)	0.53269 (11)	0.95654 (15)	0.0364 (10)	
H4D	0.7156 (2)	0.5065 (10)	0.9274 (11)	0.055*	
H4C	0.6772 (12)	0.5408 (3)	0.9514 (3)	0.055*	
O5	0.27701 (15)	0.45872 (11)	0.63870 (16)	0.0354 (10)	
H5C	0.2514 (9)	0.46011 (12)	0.6665 (10)	0.053*	
H5D	0.3138 (13)	0.4488 (4)	0.6669 (10)	0.053*	
O6	0.4078 (2)	0.4460 (3)	0.7120 (2)	0.102 (2)	
H6C	0.426 (3)	0.438 (3)	0.7536 (19)	0.154*	
H6D	0.437 (3)	0.450 (3)	0.688 (4)	0.154*	
Cl4	0.0446 (9)	0.6580 (5)	0.7868 (11)	0.136 (6)	0.347 (6)
Cl5	−0.0603 (3)	0.5963 (3)	0.7014 (4)	0.054 (2)	0.347 (6)
C99	−0.0324 (12)	0.6561 (4)	0.7228 (18)	0.034 (7)	0.347 (6)
H99A	−0.0278	0.6724	0.6752	0.041*	0.347 (6)
H99B	−0.0666	0.6734	0.7447	0.041*	0.347 (6)
C98	0.0000	0.5903 (11)	0.7500	0.051 (9)	0.306 (12)
H98A	−0.030 (7)	0.569 (4)	0.773 (8)	0.061*	0.306 (12)
Cl5A	−0.0622 (3)	0.6260 (3)	0.6985 (4)	0.044 (3)	0.306 (12)

### Atomic displacement parameters (Å^2^)

**Table:**

	*U*^11^	*U*^22^	*U*^33^	*U*^12^	*U*^13^	*U*^23^
Pt1	0.02001 (9)	0.01445 (9)	0.01693 (8)	0.00236 (10)	0.00529 (6)	0.00098 (10)
P1	0.0186 (6)	0.0153 (7)	0.0196 (6)	0.0014 (5)	0.0052 (5)	0.0007 (5)
P2	0.0236 (6)	0.0191 (6)	0.0188 (6)	0.0007 (6)	0.0076 (5)	0.0005 (6)
P3	0.0214 (6)	0.0158 (6)	0.0174 (5)	0.0019 (6)	0.0059 (5)	0.0006 (6)
P4	0.0230 (7)	0.0222 (7)	0.0259 (7)	−0.0038 (6)	0.0066 (5)	−0.0022 (6)
Cl1	0.0385 (7)	0.0222 (7)	0.0237 (6)	0.0146 (6)	0.0110 (5)	0.0054 (5)
Cl2	0.0316 (7)	0.0424 (9)	0.0283 (7)	0.0106 (6)	0.0045 (5)	−0.0028 (6)
O1	0.0274 (19)	0.033 (2)	0.0256 (17)	−0.0057 (16)	0.0056 (14)	−0.0026 (16)
C1	0.022 (2)	0.019 (3)	0.023 (2)	−0.001 (2)	0.007 (2)	−0.003 (2)
C2	0.024 (3)	0.025 (3)	0.020 (2)	0.001 (2)	0.012 (2)	0.003 (2)
C3	0.020 (2)	0.014 (3)	0.028 (3)	0.001 (2)	0.006 (2)	−0.001 (2)
C4	0.022 (2)	0.013 (2)	0.020 (2)	0.004 (2)	0.0045 (19)	0.001 (2)
C5	0.022 (2)	0.013 (2)	0.023 (2)	0.004 (2)	0.0049 (19)	−0.001 (2)
C6	0.025 (3)	0.019 (3)	0.023 (2)	0.000 (2)	0.004 (2)	0.000 (2)
C7	0.025 (3)	0.024 (3)	0.018 (2)	0.000 (2)	0.0078 (19)	0.002 (2)
C8	0.027 (3)	0.026 (3)	0.029 (3)	0.003 (2)	0.001 (2)	0.002 (2)
C9	0.027 (3)	0.032 (4)	0.034 (3)	0.010 (2)	−0.003 (2)	0.005 (3)
C10	0.025 (3)	0.051 (4)	0.026 (3)	−0.001 (3)	0.002 (2)	0.002 (3)
C11	0.040 (3)	0.026 (3)	0.028 (3)	−0.004 (3)	0.009 (2)	0.004 (2)
C12	0.019 (3)	0.027 (3)	0.027 (3)	0.004 (2)	0.001 (2)	0.006 (2)
C13	0.024 (3)	0.018 (3)	0.023 (2)	0.000 (2)	0.010 (2)	0.001 (2)
C14	0.037 (3)	0.031 (3)	0.027 (3)	−0.001 (3)	0.013 (2)	0.004 (2)
C15	0.044 (3)	0.032 (3)	0.040 (3)	0.004 (3)	0.025 (3)	0.012 (3)
C16	0.046 (4)	0.025 (3)	0.054 (3)	−0.002 (3)	0.027 (3)	0.008 (3)
C17	0.032 (3)	0.024 (3)	0.051 (3)	−0.004 (2)	0.014 (3)	−0.003 (3)
C18	0.031 (3)	0.021 (3)	0.034 (3)	−0.002 (2)	0.014 (2)	0.004 (2)
C19	0.020 (2)	0.020 (3)	0.018 (2)	0.003 (2)	0.0057 (17)	0.001 (2)
C20	0.032 (3)	0.018 (3)	0.024 (2)	0.001 (2)	0.010 (2)	−0.002 (2)
C21	0.025 (3)	0.028 (3)	0.034 (3)	−0.005 (2)	0.009 (2)	−0.009 (3)
C22	0.029 (3)	0.027 (3)	0.033 (3)	0.001 (3)	0.016 (2)	−0.001 (3)
C23	0.026 (3)	0.019 (3)	0.024 (2)	0.006 (2)	0.011 (2)	0.000 (2)
C24	0.020 (2)	0.014 (2)	0.023 (2)	0.001 (2)	0.0052 (19)	0.002 (2)
C25	0.020 (2)	0.015 (3)	0.021 (2)	0.001 (2)	0.0079 (19)	0.003 (2)
C26	0.027 (3)	0.026 (3)	0.018 (2)	0.000 (2)	0.005 (2)	0.003 (2)
C27	0.033 (3)	0.035 (3)	0.024 (3)	−0.001 (3)	0.002 (2)	−0.006 (2)
C28	0.026 (3)	0.029 (3)	0.032 (3)	−0.003 (2)	0.004 (2)	−0.008 (2)
C29	0.018 (3)	0.021 (3)	0.042 (3)	−0.006 (2)	0.008 (2)	−0.004 (2)
C30	0.025 (3)	0.027 (3)	0.026 (3)	0.000 (2)	0.006 (2)	−0.001 (2)
C31	0.028 (3)	0.030 (3)	0.025 (3)	−0.002 (2)	0.002 (2)	0.000 (2)
C32	0.034 (3)	0.028 (3)	0.036 (3)	−0.008 (3)	0.007 (2)	−0.002 (3)
C33	0.022 (3)	0.035 (3)	0.036 (3)	−0.007 (2)	0.004 (2)	0.004 (3)
C34	0.029 (3)	0.044 (4)	0.032 (3)	−0.004 (3)	0.008 (2)	0.008 (3)
C35	0.030 (3)	0.041 (4)	0.046 (3)	−0.001 (3)	0.015 (2)	−0.010 (3)
C36	0.033 (3)	0.031 (3)	0.045 (3)	−0.007 (3)	0.015 (2)	−0.011 (3)
C37	0.027 (3)	0.024 (3)	0.025 (3)	−0.007 (2)	0.005 (2)	−0.003 (2)
C38	0.061 (4)	0.038 (4)	0.031 (3)	0.013 (3)	0.010 (3)	−0.001 (3)
C39	0.047 (3)	0.045 (4)	0.030 (3)	0.017 (3)	0.009 (2)	0.002 (3)
C40	0.034 (3)	0.018 (3)	0.069 (4)	0.003 (2)	0.021 (3)	0.003 (3)
C41	0.043 (3)	0.035 (3)	0.040 (3)	−0.010 (3)	0.013 (3)	−0.018 (3)
C42	0.039 (3)	0.030 (3)	0.037 (3)	−0.012 (3)	0.003 (2)	−0.006 (3)
O3	0.041 (2)	0.041 (2)	0.042 (2)	0.0051 (19)	0.0187 (17)	0.0057 (19)
O4	0.043 (2)	0.032 (2)	0.036 (2)	−0.0009 (18)	0.0141 (16)	−0.0039 (18)
O5	0.040 (2)	0.039 (2)	0.0301 (19)	0.0009 (18)	0.0133 (15)	−0.0003 (18)
O6	0.036 (3)	0.245 (7)	0.027 (2)	−0.003 (4)	0.0055 (19)	−0.010 (4)
Cl4	0.198 (13)	0.106 (8)	0.126 (10)	−0.103 (8)	0.089 (7)	−0.088 (8)
Cl5	0.054 (4)	0.065 (4)	0.047 (3)	−0.016 (4)	0.020 (3)	−0.018 (4)
C99	0.040 (9)	0.034 (11)	0.033 (10)	−0.004 (8)	0.020 (7)	0.002 (8)
C98	0.051 (18)	0.058 (19)	0.040 (16)	0.000	0.000 (14)	0.000
Cl5A	0.037 (3)	0.054 (7)	0.037 (3)	0.009 (5)	−0.001 (2)	0.013 (5)

### Geometric parameters (Å, °)

**Table:**

Pt1—P1	2.2078 (12)	C21—H21B	0.9900
Pt1—P2	2.3151 (11)	C22—C23	1.519 (6)
Pt1—P3	2.3215 (10)	C22—H22A	0.9900
Pt1—Cl1	2.3563 (11)	C22—H22B	0.9900
P1—C3	1.806 (4)	C23—C24	1.532 (5)
P1—C1	1.815 (4)	C23—H23A	0.9900
P1—C5	1.820 (4)	C23—H23B	0.9900
P2—C13	1.823 (4)	C24—H24A	0.9900
P2—C7	1.838 (4)	C24—H24B	0.9900
P2—C2	1.849 (4)	C25—C26	1.536 (5)
P3—C19	1.820 (4)	C25—C30	1.536 (5)
P3—C4	1.842 (4)	C25—H25A	1.0000
P3—C25	1.847 (4)	C26—C27	1.523 (6)
P4—O1	1.494 (3)	C26—H26A	0.9900
P4—C6	1.805 (4)	C26—H26B	0.9900
P4—C31	1.820 (5)	C27—C28	1.514 (6)
P4—C37	1.837 (5)	C27—H27A	0.9900
Cl2—O6	3.170 (4)	C27—H27B	0.9900
Cl2—C5	3.654 (4)	C28—C29	1.513 (5)
Cl2—C3	3.755 (4)	C28—H28A	0.9900
O1—O5	2.740 (4)	C28—H28B	0.9900
C1—C2	1.539 (5)	C29—C30	1.527 (5)
C1—H1B	0.9900	C29—H29A	0.9900
C1—H1C	0.9900	C29—H29B	0.9900
C2—H2A	0.9900	C30—H30A	0.9900
C2—H2B	0.9900	C30—H30B	0.9900
C3—C4	1.537 (5)	C31—C32	1.538 (6)
C3—H3A	0.9900	C31—C36	1.540 (6)
C3—H3B	0.9900	C31—H31A	1.0000
C4—H4A	0.9900	C32—C33	1.520 (6)
C4—H4B	0.9900	C32—H32A	0.9900
C5—C6	1.532 (5)	C32—H32B	0.9900
C5—H5A	0.9900	C33—C34	1.516 (6)
C5—H5B	0.9900	C33—H33A	0.9900
C6—H6A	0.9900	C33—H33B	0.9900
C6—H6B	0.9900	C34—C35	1.517 (6)
C7—C8	1.520 (6)	C34—H34A	0.9900
C7—C12	1.523 (6)	C34—H34B	0.9900
C7—H7A	1.0000	C35—C36	1.525 (6)
C8—C9	1.545 (5)	C35—H35A	0.9900
C8—H8A	0.9900	C35—H35B	0.9900
C8—H8B	0.9900	C36—H36A	0.9900
C9—C10	1.517 (6)	C36—H36B	0.9900
C9—H9A	0.9900	C37—C42	1.521 (6)
C9—H9B	0.9900	C37—C38	1.532 (6)
C10—C11	1.526 (6)	C37—H37A	1.0000
C10—H10A	0.9900	C38—C39	1.531 (6)
C10—H10B	0.9900	C38—H38A	0.9900
C11—C12	1.516 (5)	C38—H38B	0.9900
C11—H11A	0.9900	C39—C40	1.503 (6)
C11—H11B	0.9900	C39—H39A	0.9900
C12—H12A	0.9900	C39—H39B	0.9900
C12—H12B	0.9900	C40—C41	1.520 (6)
C13—C18	1.534 (5)	C40—H40A	0.9900
C13—C14	1.535 (5)	C40—H40B	0.9900
C13—H13A	1.0000	C41—C42	1.520 (6)
C14—C15	1.530 (6)	C41—H41A	0.9900
C14—H14A	0.9900	C41—H41B	0.9900
C14—H14B	0.9900	C42—H42A	0.9900
C15—C16	1.515 (6)	C42—H42B	0.9900
C15—H15A	0.9900	O3—H3C	0.8017
C15—H15B	0.9900	O3—H3D	0.8847
C16—C17	1.536 (6)	O4—H4D	0.9154
C16—H16A	0.9900	O4—H4C	0.7198
C16—H16B	0.9900	O5—O6	2.746 (5)
C17—C18	1.519 (6)	O5—H5C	0.7892
C17—H17A	0.9900	O5—H5D	0.8623
C17—H17B	0.9900	O6—H6C	0.80 (4)
C18—H18A	0.9900	O6—H6D	0.81 (7)
C18—H18B	0.9900	Cl4—C99	1.750 (9)
C19—C20	1.535 (5)	Cl5—C99	1.790 (10)
C19—C24	1.542 (6)	Cl5—H98A	1.51 (14)
C19—H19A	1.0000	C99—H99A	0.9900
C20—C21	1.521 (5)	C99—H99B	0.9900
C20—H20A	0.9900	C98—H98A^i^	0.989 (10)
C20—H20B	0.9900	C98—Cl5A^i^	1.732 (19)
C21—C22	1.510 (6)	C98—Cl5A	1.732 (19)
C21—H21A	0.9900	C98—H98A	0.989 (10)
			
P1—Pt1—P2	84.93 (4)	C19—C20—H20B	109.3
P1—Pt1—P3	86.57 (4)	H20A—C20—H20B	108.0
P2—Pt1—P3	167.17 (4)	C22—C21—C20	111.7 (4)
P1—Pt1—Cl1	176.99 (4)	C22—C21—H21A	109.3
P2—Pt1—Cl1	92.85 (4)	C20—C21—H21A	109.3
P3—Pt1—Cl1	95.26 (4)	C22—C21—H21B	109.3
C3—P1—C1	112.32 (19)	C20—C21—H21B	109.3
C3—P1—C5	106.1 (2)	H21A—C21—H21B	107.9
C1—P1—C5	102.45 (19)	C21—C22—C23	110.7 (4)
C3—P1—Pt1	109.77 (14)	C21—C22—H22A	109.5
C1—P1—Pt1	106.73 (15)	C23—C22—H22A	109.5
C5—P1—Pt1	119.40 (14)	C21—C22—H22B	109.5
C13—P2—C7	105.74 (19)	C23—C22—H22B	109.5
C13—P2—C2	107.4 (2)	H22A—C22—H22B	108.1
C7—P2—C2	106.4 (2)	C22—C23—C24	110.7 (3)
C13—P2—Pt1	111.21 (14)	C22—C23—H23A	109.5
C7—P2—Pt1	119.04 (14)	C24—C23—H23A	109.5
C2—P2—Pt1	106.54 (13)	C22—C23—H23B	109.5
C19—P3—C4	105.7 (2)	C24—C23—H23B	109.5
C19—P3—C25	106.02 (19)	H23A—C23—H23B	108.1
C4—P3—C25	105.06 (19)	C23—C24—C19	110.5 (3)
C19—P3—Pt1	118.86 (13)	C23—C24—H24A	109.6
C4—P3—Pt1	106.41 (13)	C19—C24—H24A	109.6
C25—P3—Pt1	113.66 (13)	C23—C24—H24B	109.6
O1—P4—C6	111.06 (19)	C19—C24—H24B	109.6
O1—P4—C31	111.98 (19)	H24A—C24—H24B	108.1
C6—P4—C31	105.1 (2)	C26—C25—C30	110.0 (3)
O1—P4—C37	110.13 (19)	C26—C25—P3	113.0 (3)
C6—P4—C37	106.6 (2)	C30—C25—P3	112.0 (3)
C31—P4—C37	111.8 (2)	C26—C25—H25A	107.1
O6—Cl2—C5	63.04 (12)	C30—C25—H25A	107.1
O6—Cl2—C3	103.47 (14)	P3—C25—H25A	107.1
C5—Cl2—C3	46.01 (9)	C27—C26—C25	110.5 (4)
P4—O1—O5	117.52 (17)	C27—C26—H26A	109.5
C2—C1—P1	107.8 (3)	C25—C26—H26A	109.5
C2—C1—H1B	110.1	C27—C26—H26B	109.5
P1—C1—H1B	110.1	C25—C26—H26B	109.5
C2—C1—H1C	110.1	H26A—C26—H26B	108.1
P1—C1—H1C	110.1	C28—C27—C26	111.5 (4)
H1B—C1—H1C	108.5	C28—C27—H27A	109.3
C1—C2—P2	112.8 (3)	C26—C27—H27A	109.3
C1—C2—H2A	109.0	C28—C27—H27B	109.3
P2—C2—H2A	109.0	C26—C27—H27B	109.3
C1—C2—H2B	109.0	H27A—C27—H27B	108.0
P2—C2—H2B	109.0	C29—C28—C27	110.7 (4)
H2A—C2—H2B	107.8	C29—C28—H28A	109.5
C4—C3—P1	110.5 (3)	C27—C28—H28A	109.5
C4—C3—Cl2	137.0 (3)	C29—C28—H28B	109.5
P1—C3—Cl2	88.84 (15)	C27—C28—H28B	109.5
C4—C3—H3A	109.6	H28A—C28—H28B	108.1
P1—C3—H3A	109.6	C28—C29—C30	111.2 (4)
C4—C3—H3B	109.6	C28—C29—H29A	109.4
P1—C3—H3B	109.6	C30—C29—H29A	109.4
Cl2—C3—H3B	98.5	C28—C29—H29B	109.4
H3A—C3—H3B	108.1	C30—C29—H29B	109.4
C3—C4—P3	111.7 (3)	H29A—C29—H29B	108.0
C3—C4—H4A	109.3	C29—C30—C25	111.9 (4)
P3—C4—H4A	109.3	C29—C30—H30A	109.2
C3—C4—H4B	109.3	C25—C30—H30A	109.2
P3—C4—H4B	109.3	C29—C30—H30B	109.2
H4A—C4—H4B	107.9	C25—C30—H30B	109.2
C6—C5—P1	115.5 (3)	H30A—C30—H30B	107.9
C6—C5—Cl2	118.8 (3)	C32—C31—C36	110.3 (4)
P1—C5—Cl2	91.79 (15)	C32—C31—P4	110.7 (3)
C6—C5—H5A	108.4	C36—C31—P4	110.1 (3)
P1—C5—H5A	108.4	C32—C31—H31A	108.5
Cl2—C5—H5A	112.9	C36—C31—H31A	108.5
C6—C5—H5B	108.4	P4—C31—H31A	108.5
P1—C5—H5B	108.4	C33—C32—C31	112.9 (4)
H5A—C5—H5B	107.5	C33—C32—H32A	109.0
C5—C6—P4	111.8 (3)	C31—C32—H32A	109.0
C5—C6—H6A	109.3	C33—C32—H32B	109.0
P4—C6—H6A	109.3	C31—C32—H32B	109.0
C5—C6—H6B	109.3	H32A—C32—H32B	107.8
P4—C6—H6B	109.3	C34—C33—C32	113.6 (4)
H6A—C6—H6B	107.9	C34—C33—H33A	108.8
C8—C7—C12	110.7 (4)	C32—C33—H33A	108.8
C8—C7—P2	112.7 (3)	C34—C33—H33B	108.8
C12—C7—P2	111.3 (3)	C32—C33—H33B	108.8
C8—C7—H7A	107.3	H33A—C33—H33B	107.7
C12—C7—H7A	107.3	C33—C34—C35	110.2 (4)
P2—C7—H7A	107.3	C33—C34—H34A	109.6
C7—C8—C9	110.7 (4)	C35—C34—H34A	109.6
C7—C8—H8A	109.5	C33—C34—H34B	109.6
C9—C8—H8A	109.5	C35—C34—H34B	109.6
C7—C8—H8B	109.5	H34A—C34—H34B	108.1
C9—C8—H8B	109.5	C34—C35—C36	110.8 (4)
H8A—C8—H8B	108.1	C34—C35—H35A	109.5
C10—C9—C8	111.7 (4)	C36—C35—H35A	109.5
C10—C9—H9A	109.3	C34—C35—H35B	109.5
C8—C9—H9A	109.3	C36—C35—H35B	109.5
C10—C9—H9B	109.3	H35A—C35—H35B	108.1
C8—C9—H9B	109.3	C35—C36—C31	113.1 (4)
H9A—C9—H9B	107.9	C35—C36—H36A	109.0
C9—C10—C11	111.6 (4)	C31—C36—H36A	109.0
C9—C10—H10A	109.3	C35—C36—H36B	109.0
C11—C10—H10A	109.3	C31—C36—H36B	109.0
C9—C10—H10B	109.3	H36A—C36—H36B	107.8
C11—C10—H10B	109.3	C42—C37—C38	110.6 (4)
H10A—C10—H10B	108.0	C42—C37—P4	113.8 (3)
C12—C11—C10	111.1 (4)	C38—C37—P4	115.6 (3)
C12—C11—H11A	109.4	C42—C37—H37A	105.2
C10—C11—H11A	109.4	C38—C37—H37A	105.2
C12—C11—H11B	109.4	P4—C37—H37A	105.2
C10—C11—H11B	109.4	C39—C38—C37	110.2 (4)
H11A—C11—H11B	108.0	C39—C38—H38A	109.6
C11—C12—C7	112.4 (4)	C37—C38—H38A	109.6
C11—C12—H12A	109.1	C39—C38—H38B	109.6
C7—C12—H12A	109.1	C37—C38—H38B	109.6
C11—C12—H12B	109.1	H38A—C38—H38B	108.1
C7—C12—H12B	109.1	C40—C39—C38	110.9 (4)
H12A—C12—H12B	107.9	C40—C39—H39A	109.5
C18—C13—C14	110.6 (4)	C38—C39—H39A	109.5
C18—C13—P2	112.9 (3)	C40—C39—H39B	109.5
C14—C13—P2	114.1 (3)	C38—C39—H39B	109.5
C18—C13—H13A	106.2	H39A—C39—H39B	108.1
C14—C13—H13A	106.2	C39—C40—C41	110.3 (4)
P2—C13—H13A	106.2	C39—C40—H40A	109.6
C15—C14—C13	111.7 (4)	C41—C40—H40A	109.6
C15—C14—H14A	109.3	C39—C40—H40B	109.6
C13—C14—H14A	109.3	C41—C40—H40B	109.6
C15—C14—H14B	109.3	H40A—C40—H40B	108.1
C13—C14—H14B	109.3	C40—C41—C42	111.1 (4)
H14A—C14—H14B	108.0	C40—C41—H41A	109.4
C16—C15—C14	111.3 (4)	C42—C41—H41A	109.4
C16—C15—H15A	109.4	C40—C41—H41B	109.4
C14—C15—H15A	109.4	C42—C41—H41B	109.4
C16—C15—H15B	109.4	H41A—C41—H41B	108.0
C14—C15—H15B	109.4	C41—C42—C37	111.6 (4)
H15A—C15—H15B	108.0	C41—C42—H42A	109.3
C15—C16—C17	110.0 (4)	C37—C42—H42A	109.3
C15—C16—H16A	109.7	C41—C42—H42B	109.3
C17—C16—H16A	109.7	C37—C42—H42B	109.3
C15—C16—H16B	109.7	H42A—C42—H42B	108.0
C17—C16—H16B	109.7	H3C—O3—H3D	102.7
H16A—C16—H16B	108.2	H4D—O4—H4C	112.1
C18—C17—C16	111.3 (4)	O1—O5—O6	114.12 (14)
C18—C17—H17A	109.4	O6—O5—H5C	113.4
C16—C17—H17A	109.4	O1—O5—H5D	105.0
C18—C17—H17B	109.4	H5C—O5—H5D	104.2
C16—C17—H17B	109.4	O5—O6—Cl2	131.45 (17)
H17A—C17—H17B	108.0	O5—O6—H6C	136 (6)
C17—C18—C13	110.1 (4)	O5—O6—H6D	118 (6)
C17—C18—H18A	109.6	Cl2—O6—H6D	106 (6)
C13—C18—H18A	109.6	H6C—O6—H6D	106 (7)
C17—C18—H18B	109.6	C99—Cl5—H98A	103 (5)
C13—C18—H18B	109.6	Cl4—C99—Cl5	112.0 (8)
H18A—C18—H18B	108.2	Cl4—C99—H99A	109.2
C20—C19—C24	109.2 (3)	Cl5—C99—H99A	109.2
C20—C19—P3	111.0 (3)	Cl4—C99—H99B	109.2
C24—C19—P3	113.8 (3)	Cl5—C99—H99B	109.2
C20—C19—H19A	107.5	H99A—C99—H99B	107.9
C24—C19—H19A	107.5	H98A^i^—C98—Cl5A^i^	97 (10)
P3—C19—H19A	107.5	H98A^i^—C98—Cl5A	124 (9)
C21—C20—C19	111.6 (3)	Cl5A^i^—C98—Cl5A	109.2 (18)
C21—C20—H20A	109.3	H98A^i^—C98—H98A	107 (10)
C19—C20—H20A	109.3	Cl5A^i^—C98—H98A	124 (9)
C21—C20—H20B	109.3	Cl5A—C98—H98A	97 (10)

Symmetry codes: (i) −*x*, *y*, −*z*+3/2.

### Hydrogen-bond geometry (Å, °)

**Table:**

*D*—H···*A*	*D*—H	H···*A*	*D*···*A*	*D*—H···*A*
O3—H3C···Cl2^ii^	0.80	2.50	3.300 (3)	175
O3—H3D···Cl2	0.88	2.43	3.290 (4)	166
O4—H4C···O3	0.73	2.16	2.866 (4)	165
O4—H4D···O5^iii^	0.92	1.82	2.730 (4)	174
O5—H5C···O1	0.80	1.94	2.740 (4)	179
O5—H5D···O6	0.87	1.92	2.746 (5)	159
O6—H6C···Cl2	0.80 (4)	2.43 (4)	3.170 (4)	155 (8)
O6—H6D···Cl2^iii^	0.81 (7)	2.40 (7)	3.200 (4)	175 (9)
C3—H3A···Cl2	0.99	2.92	3.755 (4)	142
C5—H5B···Cl2	0.99	2.72	3.655 (4)	157

Symmetry codes: (ii) −*x*+1, −*y*+1, −*z*+2; (iii) −*x*+1, *y*, −*z*+3/2.
